# Avian data from Kenyir rainforest trail

**DOI:** 10.1016/j.dib.2018.11.119

**Published:** 2018-11-28

**Authors:** Bryan Raveen Nelson, Gertrude David, Ahmad Fakhrurrazi Mokhtar, Mazrul Aswady Mamat, Abdul Jalil Abdul Rahman

**Affiliations:** a Institute of Tropical Biodiversity and Sustainable Development (ITBSD), Universiti Malaysia Terengganu, 21030 Kuala Nerus, Terengganu, Malaysia; b Central Laboratory (MB), Universiti Malaysia Terengganu, 21030 Kuala Nerus, Terengganu, Malaysia; c School of Marine and Environmental Sciences (PPSMS), Universiti Malaysia Terengganu, 21030 Kuala Nerus, Terengganu, Malaysia; d Bird Group Taman Negara (BGTN), Kuala Tahan, 27000 Jerantut, Pahang, Malaysia

## Abstract

This data article is constructed using avian (bird) counts from a recently identified trail in Kenyir rainforest, East Peninsular Malaysia. Avian chirps and naked eye visual were simultaneously used to locate the birds. After visual binocular and digital image inspection, identification of avian species were carried out using reference books. Data tabulation are divided by monsoon seasons and months before interpret using Shannon and Evenness indices. The highlights like feeding guilds, nativity, iconic species and statuses in the wild are presented with the data to increase its value. Within these, a total of 457 avian individuals from 36 avian family groups were recorded from which, 25 of these avian species occur as near threatened, vulnerable, endangered and critically endangered in the wild. Having these, the tabulated data becomes a calendar for seasonal availability of avian species which considers the 1.0 km trail suitable for bird watching, scientific study and ecotourism purposes.

**Specifications table**

**Table:** 

*Criteria*	*Descriptions*
*Subject area*	*Biology*
*More specific subject area*	*Bioscience and Biodiversity*
*Type of data*	Tables
*How data was acquired*	*Visual using binocular (7* × *50 = 350 mm), DSLR camera capture using 400 mm lens, cellular GPS (Google Maps) and Paleontological Statistics Software Package (PAST) v.3*
*Data format*	*Raw and partially analyzed*
*Experimental factors*	*1.0 km trail with 300 m horizontal and 30 m vertical visibility, time dedication, daily frequency, lunar calendar, occurrence by chance, clarity of avian chirping, human naked-eye visibility, avian identification technique, (7* × *50 = 350 mm) binocular lens visibility and DSLR camera 400 mm zoom lens visibility and camera settings for image clarity*
*Experimental features*	*Avian species and family counts within 5 months, monsoon seasons, abundance pool, avian statuses in the wild, possible feeding guilds and diversity estimations using Shannon and Evenness Indices*
*Data source location*	*Hulu Terengganu, Terengganu, East Peninsular Malaysia*
	*Pengkalan Utama Road*: N5° 8′33.73″, E 102°45′37.63″ - N 5° 8′53.31″, E 102°45′52.74″
*Data accessibility*	*All raw data are available within this article*
*Related research article*	*Unpublished data*

**Value of the data**

• The data becomes an avian species calendar at Kenyir rainforest, Hulu Terengganu.
• This tabulated data highlights avitourism potential at the newly identified 1.0 km trail.
• The avian species records becomes an electronic database that supports other findings especially when applied towards single-species or iconic species spatial-temporal distribution.
• Availability of this data reduces study-overlaps at the national park area within states Kelantan, Terengganu and Pahang in Peninsular Malaysia.

## 1 Data

The data is constructed using 457 avian individuals sighted at the Kenyir rainforest trail. In this 5 months opportunistic find, a total of avian 113 species from 36 bird family groups were transformed into a calendar to indicate seasonal avian occurrences. Additional information like statuses, feeding guilds, locality and diversity measurements (Shannon and Evenness indices) are compiled with the data to highlight iconic and treasured avian species available in Kenyir rainforest.

## 2 Experimental design, materials and methods

Experience in Kenyir rainforest were used as basis for location selection, transect construction and avian watching period [1], [2]. Pilot attempts were carried out along Pengkalan Utama road during June 2017 to identify possible birding trail having optimum visibility and with highest avian records. Hence, the present avian database construction begun in July 2017 at a 1.0 km stretch on the aforementioned road. In the (1000 m × 2.5 m) 2500 m^2^ transect, avian counting were carried out once-per-month for period of 5 months in two daily frequencies between 0700–1000 and 1630–1900. Avian chirping and naked-eye visual were used to locate avian positions whereas binoculars (7 × 50 = 350 mm) and DSLR camera with 400 mm lens were used to improve data capture. Avian species were identified using coloration and patterns on the crown, nape, throat, wing, rump, belly, breast and tail from guide books [3], [4] and experiences [5] before presented as family groups with feeding guild, statuses in the wild and locality (Table 1). With consideration to good visibility and dry conditions during Southwest monsoon, iconic avian counting had better yield than it were during the wet and gloomy Inter-monsoon and Northeast monsoon periods (Table 2). Additional information like total family and species counts are presented together with biodiversity measurements prepared using Shannon and Evenness indices from Paleontological Statistics Software Package - PAST v.3 (Table 3).

**Table 1:** Kenyir avian calendar tabulated as family, counts, statuses in the wild, feeding guilds and locality.

Avian family	Southwest monsoon												Intermonsoon				Northeast monsoon			
	July				August				September				October				November			
	C	S	G	L	C	S	G	L	C	S	G	L	C	S	G	L	C	S	G	L
Accipitridae	1	LC	1	N					1	LC	1,5	N					1	LC	1,5	N
Aegithinidae	5	LC,NT	1	N,I					4	LC,NT	1	N,I					1	NT	1	I
Alcedinidae	4	LC	1,5	B,N	2	LC	1,5	N												
Apodidae	14	LC,NT	1	N,I					12	LC	1	I	2	LC	1	I				
Bucerotidae	8	LC,NT,CR	1,2	N,I	1	LC	1,2	I	2	NT	1,2	I								
Campephagidae	4	LC,NT	1,2	I																
Chloropseidae	3	NT	1,2,6	I	3	NT	1,2,6	I	4	NT,VU	1,2,6	I,E					3	NT	1,2,6	I
Cisticolidae	8	LC	1,6	N,I					2	LC	1,6	N								
Columbidae	27	LC	1,2,3,4	N,I	41	LC	1,2,3	N	8	LC	1,2,3	N	5	LC	1,3	N	3	LC	1,3	N
Coraciidae	1	LC	1	N																
Corvidae	4	LC,NT	1,2	I																
Cuculidae	4	LC	1,2,3	N,I					2	LC	1	I	1	LC	1	I				
Dicaeidae	11	LC	1,2,3,4,6	N,I	1	LC	1,2,3,4,6	I	5	LC	1,2,3,4,6	I								
Dicruridae	4	LC	1,6	N									1	LC	1,6	N				
Estrildidae	13	LC	1,2,3,4	N																
Eurylaimidae	8	LC,NT	1,2,4	I																
Hemiprocnidae	2	LC	1,5	I					1	LC	1,5	I	2	LC	1,5	I	6	LC	1,5	I
Hirunidae	6	LC	1	E																
Irenidae	8	LC	1,2,6	N					1	LC	1,2,6	N								
Megalaimidae	7	NE,LC,NT	1,2	I	1	NT	1,2	I												
Meropidae	7	LC	1	N,I																
Monarchidae	2	LC	1	N																
Motacillidae	2	LC	1,3,4	N																
Musicapidae	11	NE,LC,NT	1,2,6	N,I	5	LC	1,2,6	N	2	LC	1,2,6	N	1	LC	1,2,6	N	1	LC	1,2,6	N
Nectariniidae	13	LC	1,2,3,4,6	N,I					2	LC	1,2,3,6	N	1	LC	1,2,3,6	N				
Oreolidae	2	LC,NT	1,2	N,I					2	LC,NT	1,2	N,I								
Passeridae																	1	LC	1,4	B
Pellorneidae	7	LC,NT	1,2	N,I,E					1	LC	1,2	N								
Phasianidae	1	NT	1,2,3,4	I																
Picidae	10	NE,LC,EN,NT	1,2,6	I,E																
Psittacidae	2	LC	2,4	I																
Pycnonotidae	51	NE,LC,NT	1,2,3,4	I,E	8	LC	1,2,3	I	14	LC,NT	1,2,3,4	I	9	LC,NT	I,2,3	I	4	LC	1,2,3	I
Stenostiridae	2	LC	1	N																
Sturnidae	12	LC,VU	1,2,3,4,6	N,I,T	4	LC	1,2,6	I	4	LC	1,2,3,4,6		1	LC	1,2,6	I				
Tephrodornithidae	14	NE,LC	1,2	N,I					1	NE	1,2	I								
Zosteropidae	2	LC	1,2,6	I																

*Note*: The avian family are sub-divided into C = count as number of individuals - nos.; S = Status in International Union for Conservation of Nature (IUCN) Red List; G = Feeding guilds and L = Locality. The IUCN status are sub-divided into NE = Not Evaluated; LC = Least Concern; NT = Near Threatened; VU = Vulnerable; EN = Endangered; CR = Critically Endangered. Feeding guilds are classified as 1 = Carnivore; 2 = Frugivore; 3 = Granivore; 4 = Herbivore; 5 = Piscivore and; 6 = Nectarivore. The Locality is described as B = Broad Occurrence; E = Endemic to Indonesia; I = Indigenous; N = Native and T = Introduced.

**Table 2:** Selected iconic avian species form Kenyir rainforest, their local names, statuses in the wild and counts.

Avian species	Local name	5-month count (nos.)
Near Threatened		
*Aegithina viridissima*	Green Iora	6
*Alcippe brunneicauda*	Brown Fulvetta	2
*Anthracoceros malayanus*	Black Hornbill	2
*Buceros rhinosceros*	Rhinoceros Hornbill	4
*Calyptomena viridis*	Green Broadbill	3
*Chloropsis cyanopogon*	Lesser Green Leafbird	12
*Enicurus ruficapillus*	Chestnut-naped Forktail	1
*Eurylaimus ochromalus*	Black-and-yellow Broadbill	3
*Hydrochous gigas*	Waterfall Swift	7
*Iole olivacea*	Buff-vented Bulbul	6
*Megalaima mystacophanos*	Red-throated Barbet	4
*Megalaima rafflesii*	Red-crowned Barbet	2
*Meiglyptes tukki*	Buff-necked woodpecker	1
*Oriolus xanthonotus*	Dark-throated Oriole	2
*Pericrocotus igneus*	Fiery Minivet	3
*Platylophus galericulatus*	Crested Jay	1
*Pycnonotus cyaniventris*	Grey-bellied Bulbul	2
*Pycnonotus eutilotus*	Puff-backed Bulbul	1
*Pycnonotus goiavier*	Yellow-vented Bulbul	19
*Pycnonotus squamatus*	Scaly-breasted Bulbul	1
*Rhizothera longirostris*	Long-billed Partridge	1
Vulnerable		
*Acridotheres javanicus*	Javan Myna	6
*Chloropsis sonnerati*	Greater Green Leafbird	1
Endangered		
*Meiglyptes tristis*	Buff-rumped Woodpecker	3
Critically Endangered		
*Rhinoplax vigil*	Helmeted Hornbill	1

*Note*: The 5-month count data for avian species are presented as number of individuals (nos).

**Table 3:** Pooled data and Shannon and Evenness indices for avian individuals counted at Kenyir rainforest.

Criteria	Southwest			Intermonsoon	Northeast monsoon
	July	August	September	October	November
Avian Family (nos.)	35	9	18	9	8
Avian Species (nos.)	102	12	33	11	9
Abundance (nos.)	280	66	68	23	20
Shannon Index	4.19	1.56	2.98	1.98	1.79
Evenness Index	0.65	0.40	0.60	0.66	0.67

*Note*: The avian family, species and total abundance are pooled by numbers (nos.).
