# Supplementary material for: Avian data from Kenyir rainforest trail
Source: Data Brief. 2018 Nov 28;21:2633–7. doi: 10.1016/j.dib.2018.11.119 (PMC6290243; doi:10.1016/j.dib.2018.11.119)
Supplement: Supplementary file 1 — Transparency document. [file mmc1.doc]

Conflict of Interest and Authorship Conformation Form

Please check the following as appropriate:

X All authors have participated in (a) conception and design, or analysis and interpretation of the data; (b) drafting the article or revising it critically for important intellectual content; and (c) approval of the final version.

X This manuscript has not been submitted to, nor is under review at, another journal or other publishing venue.

X The authors have no affiliation with any organization with a direct or indirect financial interest in the subject matter discussed in the manuscript.

The following authors have affiliations with organizations with direct or indirect financial interest in the subject matter discussed in the manuscript:

Author’s name Affiliation
